# Genotype mutations and phenotypes of 30 cases with epilepsy related to fever sensitivity in children

**DOI:** 10.3389/fneur.2026.1766799

**Published:** 2026-04-09

**Authors:** Yanping Wang, Lin Zhang, Jingbo Ma, Miao Jing, Ying Hua, Yanshan Liu, Xiaochun Fan

**Affiliations:** 1 Department of Neurology, Affiliated Children’s Hospital of Jiangnan University (Wuxi Children’s Hospital), Wuxi, Jiangsu Province, China; 2 Department of Pediatric Laboratory, Affiliated Children’s Hospital of Jiangnan University (Wuxi Children’s Hospital), Wuxi, Jiangsu Province, China; 3 Department of Emergency, Wuxi No.2 People’s Hospital, Wuxi, Jiangsu Province, China

**Keywords:** epilepsy related to fever sensitivity, gene mutation, KIAA2022, PCDH19, SCN1A

## Abstract

**Objective:**

To explore the clinical features and molecular genetic characteristics of epilepsy related to fever sensitivity caused by various types of gene mutations, and to analyze the relationships of genotype and clinical phenotype with clinical treatment efficacy.

**Methods:**

This retrospective study was conducted on 30 cases of children with abnormal genetic testing related to febrile sensitivity epilepsy treated in Wuxi Children’s Hospital between June 2016 and April 2023. All 61 children who met the inclusion criteria underwent whole exome sequencing (WES); clinical features were compared between the 30 gene-positive patients and the 31 gene-negative patients. Genetic testing results and clinical data of the 30 positive cases were summarized and the children were divided into “effective” and “ineffective” groups according to the efficacy of clinical treatment for comparisons.

**Results:**

Among the 30 gene-positive children, the onset of epilepsy occurred early, with 20 cases occurring within 1 year after birth, and 17 cases having developmental delay. Thirty cases of pathogenic genes related to epilepsy were detected with mutations in the *SCN1A* gene (13 cases), *PCDH19* (4 cases), *ADGRV1* (3 cases), and *CACNB4* (2 cases) as well as one case each of mutations in *SCN2A*, *PRRT2*, *CACNA1A*, *CACNA1E*, *CACNA1H*, *KCNA2*, *CHD2*, and *KIAA2022*, which was identified as a novel gene mutation related to epilepsy. The final diagnosis was 11 cases (36.7%) of Dravet syndrome, four cases (13.3%) of *PCDH19*-related epilepsy, four cases (13.3%) of generalized epilepsy with febrile seizures plus, one case (3.3%) of Epilepsy with myoclonic-atonic seizures, two cases (6.7%) of focal epilepsy, and eight cases (26.7%) of other types of epilepsy. There were differences between the ‘effective’ and ‘ineffective’ groups in the different pathogenic levels of American College of Medical Genetics and Genomics (ACMG) classification, mutation type (gene) and the onset age group (≤1 year group) (*p* < 0.05), while there were no differences in sex, presence or absence of status epilepticus, and ion channel efficacy (*p* > 0.05). Comparison between the positive and negative groups revealed that patients in the positive group had a significantly earlier age at onset (*p* < 0.05), a higher frequency of status epilepticus (*p* < 0.05), and a higher rate of developmental delay after onset (*p* < 0.001).

**Conclusion:**

Febrile sensitivity-related epilepsy in children is primarily caused by *SCN1A*, *PCDH19*, and *ADGRV1* mutations, manifesting mainly as Dravet syndrome and PCDH19-related epilepsy. The novel *KIAA2022* mutation expands the gene spectrum of this condition. Early age at onset, status epilepticus, and developmental delay are clinical red flags for genetic etiology, supporting early genetic testing in infants ≤1 year to guide precision treatment.

## 1 Introduction

Epilepsy is one of the most common neurological diseases in childhood, with a prevalence ranging from 4% to 7% in China (1). Early and accurate detection of childhood epilepsy is critical for guiding precise clinical treatment and strategies to control symptoms, and enhancing the long-term prognosis for the affected child. The etiology of the epilepsy is multifaceted, factors including structural, genetic, metabolic, infectious, immune, and idiopathic are commonly observed in clinical practice. However, some forms of epilepsy are not easy to be distinguished from febrile seizures in the initial stages. As the condition progresses, the affected child may gradually experience episodes of low fever or non-febrile seizures, yet the sensitivity to fever often remains. Diagnosis of the corresponding epilepsy features typically occurs when the child reaches a certain age.

In clinical practice, epilepsy closely associated with febrile seizures is referred to as epilepsy related to fever sensitivity, encompassing a spectrum of conditions that covers various types of epilepsy and epilepsy syndromes (2). Notable types of epilepsy related to fever sensitivity include Dravet syndrome (DS), *PCDH19* gene-related epilepsy, generalized epilepsy with febrile seizures plus (GEFS+), and Epilepsy with myoclonic-atonic seizures (EMAS) (3, 4).

The rapid development of second-generation gene sequencing technology, coupled with its application in clinical practice, has greatly expedited the identification of an increasing number of gene mutations linked to febrile-sensitive epilepsy. The identified mutations exhibit distinct phenotypes and genetic heterogeneity. Currently, domestic research on epilepsy related to fever sensitivity has predominatly focused on case reports, or studies with small sample sizes, and reviews. However, there is a noticeable dearth of research addressing the correlation between genotype and phenotype in this context.

In the present study, we conducted an analysis of both the clinical and gene mutation characteristics in 30 children with epilepsy related to fever sensitivity. The study cohort consisted of individuals who sought medical attention at Wuxi Children’s Hospital from June 2016 to April 2023. Furthermore, we compared the clinical features of these 30 genetically positive patients with those of 31 genetically negative patients to identify potential early clinical predictors of genetic etiology. Our findings in the current study aim to contribute novel insights and potential strategies for the early diagnosis and precise treatment of this particular form of epilepsy.

## 2 Materials and Methods

### 2.1 Inclusion and exclusion criteria

This study was approved by the Ethics Committee of the Affiliated Children’s Hospital of Jiangnan University (approved number: WXCH2023-06-048). Cases met the following inclusion criteria were included in the study: (1). The nature of the first attack meets the diagnostic criteria for febrile seizures (5); Febrile seizures in children aged 6 months to 5 years is a convulsion with fever occurring within 24 h (rectal temperature ≥38.5 °C, axillary temperature ≥38 °C) without evidence of central nervous system infection or other causes of convulsion. (2). Fever easily induces attacks. (3). Disease meets the diagnostic criteria for epilepsy and epilepsy syndromes according to the International League Against Epilepsy (ILAE) 2017 classification (6). (4). Both the patient and the guardian agree and sign the informed consent form.

The following criteria were applied for exclusion: (1). A history of intracranial infection, intracranial occupation or trauma. (2). Combined with acute and chronic systemic diseases (such as heart, liver, lung, kidney diseases, metabolic diseases and rheumatic diseases, etc). (3). A confirmed diagnosis of a major psychiatric disorder (e.g., schizophrenia, autism spectrum disorder) that preceded the onset of epilepsy and was considered an independent comorbidity rather than a manifestation of the epilepsy syndrome. (4). Presence of severe infection or acute systemic illness at the time of initial genetic testing or phenotypic assessment, which could independently affect neurological evaluation.

### 2.2 Clinical data collection

A research team composed of two neurology specialists developed an information collection questionnaire to record all relevant clinical data of all children enrolled in the study, including age, sex, family history, growth and development history, age at onset, type of epilepsy attack, attack frequency, epilepsy syndrome, neurological signs, related scale examination, video EEG detection, cranial MRI, gene report, types of antiepileptic drugs, and clinical treatment efficacy. Clinical data were collected for all 61 patients who met the inclusion criteria and underwent WES. Based on genetic testing results, patients were divided into a genetically positive group (*n* = 30) and a genetically negative group (*n* = 31). Clinical features at onset, including age at first seizure, ccdzamily history, status epilepticus, developmental status, and EEG/MRI findings, were compared between the two groups.

### 2.3 Gene testing methods

Peripheral blood samples were from each child (3 mL) and parents (2 mL). Genomic DNA was extracted from the peripheral blood samples using the FlexiGene DNA kit (Qiagen) according to the manufacturer’s instructions and stored at −20 °C prior to use. Genomic DNA sequencing was performed on the Illumina platform using GRCH37/hg19 as a reference gene, and quality control was performed on the results. Possible copy number variations were analyzed, gene-related annotation and protein damage analysis were performed, and mutation sites for verification by the first generation were selected in combination with relevant information such as clinical symptoms, gene inheritance modes, mutation frequency, and sequencing depth. All rare variants filtered were annotated according to the standards and guidelines recently recommended by the American College of Medical Genetics and Genomics (ACMG) (7), and divided into five categories: pathogenic, likely pathogenic, uncertain significance, benign, and likely benign. For suspected sites, Sanger sequencing was used to verify the sequences in the child and parents, and false positive sites identified in the second-generation sequencing were excluded through first-generation sequencing verification. The age at blood collection for genetic testing and the interval from first seizure to testing were recorded for all 61 patients. For the entire cohort, the median age at genetic testing was 3.4 years (range: 0.8–13.5 years), and the median interval from first seizure to testing was 2.1 years (range: 0.1–12.8 years). For the genetically positive group (*n* = 30), the median age at testing was 3.2 years (range: 0.8–13.5 years), with a median interval of 2.1 years (range: 0.1–12.8 years). For the genetically negative group (*n* = 31), the median age at testing was 3.7 years (range: 1.3–9.3 years), with a median interval of 2.2 years (range: 0.7–7.7 years).

### 2.4 Antiepileptic drug classification

The efficacy of antiepileptic drugs was divided into the following categories: Clinical control: No epileptic seizures for 6 months after treatment; Effective: The frequency of convulsions is reduced by more than 50% 6 months after treatment; Ineffective: The frequency of seizures is reduced by less than 50% 6 months after treatment. In this study, we classified the first two are classified categories as the “effective group,” and the latter as the “ineffective group.”

### 2.5 Follow-up

The follow-up was conducted through a combination of outpatient visits and phone calls. The follow-up content included: the use of antiepileptic drugs, changes in the type and frequency of epileptic seizures, seizure control, mental and motor development, etc. Follow-up was continued for at least 6 months for all patients.

### 2.6 Statistical analysis

All statistical analysis was perfumed using SPSS26.0 software. Measurement data are expressed as mean ± standard deviation, count data were described by the number of cases and percentage (%), comparison between groups of categorical variables was performed using the chi-square test or Fisher’s exact probability method. *p* < 0.05 was considered to indicate statistical significance.

## 3 Results

### 3.1 Overview of WES results

A total of 61 children with epilepsy related to fever sensitivity who visited our pediatric department from June 2016 to April 2023 and met the inclusion criteria, were collected for whole exome sequencing (WES), among which 30 cases had positive genetic abnormalities, and were used for further evaluation.

With WES, we identified 14 cases of sodium ion-related gene mutations, including 13 cases with *SCN1A* gene mutations were detected, of which 53.8% (7/13 cases) of the mutation had not been reported before. The seven novel mutation consisted two missense mutations, one nonsense mutation, and four frameshift mutations. One case with *SCN2A* gene mutation was detected. We also detected four cases with *PCDH19* mutations, including two novel mutations. Five cases with calcium ion-related genes mutations were identified, including one with a deletion encompassing exon1-14 of *CACNB4*, who also had developmental delay. One with a novel missense mutation in *CACNB4*, who was prone to status epilepticus. Another case with a missense mutation in *CACNA1H*, who showed epilepsy with myoclonic atonic seizure EMAS. The other two cases had mutations in *CACNA1E* and *CACNA1A*, respectively, both of whom achieved good epilepsy control after treatment. We also identified novel mutations in *ADGRV1* (3 cases), *PRRT2* (overall duplication in 1 case), *CHD2* (frameshift mutation in 1 case), *KCNA2* (missense mutation in 1 case), and *KIAA2022* (missense mutation c.2667G>A in 1 case). The *KIAA2022* missense mutation was identified as a novel pathogenic mutation.

For all 30 probands with positive genetic findings, Sanger sequencing was performed in available parental samples to determine the mutation origin. Parental samples were available for 27 of the 30 cases; among these, 21 mutations were confirmed as *de novo* and six were inherited (one maternal *SCN2A*, two maternal *ADGRV1*, one paternal *CACNB4*, one paternal *CACNA1E*, and one paternal *ADGRV1*). In the three cases where parental samples were unavailable (Patients 3, 7, and 22), mutation origin could not be definitively determined, although clinical histories suggested *de novo* origins. Detailed mutation origin information for each case is provided in Table 1.

**Table 1:** Genotype and phenotype of 30 patients with epilepsy related to fever sensitivity.

Patient No.	Sex	Onset Age	Gene	Age at genetic testing	Variant	ACMG classification (reported)	Mutation origin	Seizure type	Status epilepticus	DD	Drugs	Clinical efficacy	Disease
1	Female	6	SCN1A	30	c.3076delG (p.V1026X)	P	De novo	GTCS, Fs, MS	Yes	Yes	VPA, LEV fever plus DZ	effective	DS
2	Male	7	SCN1A	30	c.5245G>T (p.G1749Ter)	P	De novo	GTCS, Fs, MS	Yes	Yes	VPA, LEV	effective	DS
3	Male	4	SCN1A	22	c.301C>T (p.R101W)	P	NA	GTCS, Fs, MS	Yes	Yes	VPA, TPM, KD, CLB	ineffective	DS
4	Male	6	SCN1A	25	c.5741_5742delAA (p.Q1914fs*30)	P	De novo	GTCS, Fs, MS	No	Yes	VPA, LEV, PER	ineffective	DS
5	Female	9	SCN1A	32	c.4985_4989delCTTTG (p.A1662Dfs*9)	P	De novo	GTCS, Fs, MS	Yes	Yes	VPA, TPM, PER	ineffective	DS
6	Male	5	SCN1A	48	c.5164A>G (p.T1722A)	P	De novo	GTCS, Fs, MS	No	Yes	LEV, TPM, KD	effective	DS
7	Female	10	SCN1A	35	c.5353A>G (p.I1785V)	P	NA	GTCS, Fs, MS	Yes	Yes	VPA, PER	effective	DS
8	Female	7	SCN1A	21	c.767_769delTCT	P	De novo	GTCS, Fs, MS	Yes	Yes	VPA, TPM, KD	ineffective	DS
9	Male	6	SCN1A	39	c.580G>A p.D194N	P	De novo	GTCS, Fs, MS	Yes	Yes	VPA, TPM, KD, LEV, CZP	ineffective	DS
10	Female	6	SCN1A	24	c.814_817delAACC (p.N272X)	P	De novo	GTCS, Fs	Yes	No	OXC, LEV	effective	GEFS+
11	Male	6	SCN1A	48	c.3755A>C (p.L1252W)	LP	De novo	GTCS, Fs, MS	Yes	Yes	VPA, LEV, TPM	effective	DS
12	Male	9	SCN1A	13	c.3733C>G (p.R1245G)	LP	De novo	GTCS, Fs, MS, A	Yes	Yes	VPA, LEV	effective	DS
13	Female	15	SCN1A	47	c.4786C>T (p.R1596C)	LP	De novo	GTCS	No	No	LEV	effective	GEFS+
14	Male	24	SCN2A	40	c.3083C>G (p.A1028G)	VUS	Maternal	GTCS	No	No	LEV	effective	GEFS+
15	Female	8	PCDH19	31	c.1703dupT (p.I569DfsTer2)	P	De novo	GTCS, Fs	Yes	Yes	VPA, OXC, LEV, CZP	ineffective	PCDH19 gene-related epilepsy
16	Female	8	PCDH19	162	c.3245_3246insGTGTC (p.S1082fs)	P	De novo	GTCS, MS	Yes	Yes	VPA, TPM, CZP, PER	ineffective	PCDH19 gene-related epilepsy
17	Female	12	PCDH19	21	PCDH19 5′UTR	P	De novo	GTCS, MS	No	No	OXC, LEV, CZP	ineffective	PCDH19 gene-related epilepsy
18	Female	8	PCDH19	9	c.1031C>T (p.P344L)	LP	De novo	GTCS, MS	No	Yes	VPA, OXC, TPM, CZP	effective	PCDH19 gene-related epilepsy
19	Female	15	CACNB4	58	Deletion of exon1-14	P	De novo	Fs → GTCS	No	Yes	VPA, fever plus DZ	effective	FsEP
20	Female	13	CACNB4	49	c.121A>G (p.T41A)	VUS	Paternal	GTCS	Yes	No	LEV	effective	EP
21	Male	12	CACNA1H	38	c.3633G>A (p.R1215H)	LP	De novo	MS, AS, A	No	Yes	VPA, CZP, LTG	effective	EMAS
22	Male	24	CACNA1A	38	c.6975_6976insCAGCAGCAG (p.A2326delinsQQQA)	VUS	NA	GTCS	No	No	LEV	effective	EP
23	Female	15	CACNA1E	48	c.352A>G (p.T118A)	VUS	Paternal	GTCS	No	No	LEV	effective	EP
24	Male	13	ADGRV1	26	c.13763G>A (p.G4588E)	LP	Maternal	GTCS	No	No	LEV, fever plus DZ	effective	GEFS+
25	Female	15	ADGRV1	17	c.9829A>T (p.N3277Y)	VUS	Maternal	GTCS	Yes	No	LEV	effective	EP
26	Male	5	ADGRV1	114	c.3748G>C (p.E1250Q)	VUS	Paternal	GTCS	No	No	LEV	effective	EP
27	Female	28	CHD2	136	c.412dupG (p.D138Gfs*2)	P	De novo	Fs	No	No	OXC, VPA, LEV, LTG	effective	FsEP
28	Male	10	PRRT2	72	Total Repeat	VUS	De novo	GTCS	No	No	LEV	effective	EP
29	Male	36	KIAA2022	68	c.2667G>A (p.W889X)	P	De novo	GTCS、A	No	No	VPA, LTG, Fever plus DZ	effective	EP
30	Male	2	KCNA2	55	c.1238T>A (p.F413Y)	P	De novo	Fs、A、MS	No	Yes	LEV, TPM, VPA	effective	EP

P, Pathogenic; LP, Likely pathogenic; VUS, Variant of uncertain significane; DD, Developmental Delay; A, Absence seizure; CZP, Clonazepam; Fs, Focal seizures; GTCS, Generalized tonic–clonic seizures; MS, Myoclonic seizures; KD, Ketogenic diet; LEV, Levetiracetam; LTG, Lamotrigine; OXC, Oxcarbazepine; PER, Perampanel TPM, Topiramate; VPA, Valproic acid. Effective drugs are underlined.

### 3.2 Clinical phenotype characteristics of the 30 selected children

The selected 30 children with epilepsy related to fever sensitivity comprised 15 males (50.0%) and 15 females (50.0%), within the age range of 1 to 11 years. The onset of epilepsy was between 2 months and 3 years after birth, with a majority of 20 cases manifesting within the first year of life. Ten children had a familial predisposition to febrile seizures, while four had a family history of epilepsy. Furthermore, seven cases were misdiagnosed as febrile seizures or had atypical clinical manifestations.

Among the 30 children, 11 cases (36.7%) were finally diagnosed as DS, four cases (13.3%) as GEFS+, four (13.3%) as *PCDH19* gene-related epilepsy, two cases (6.7%) as focal epilepsy, one case (3.3%) as EMAS, and eight cases (26.7%) as other types of epilepsy. Predominant clinical seizure manifestations included myoclonic seizures, generalized tonic–clonic seizures, spasm seizures, absence seizures, and atonic seizures. Notably, 19 cases exhibited multiple seizure forms, 14 experienced cluster seizures occurring intermittently from several to more than 10 times per day, and 14 encountered episodes of status epilepticus. Furthermore, 15 children demonstrated a condition of refractory epilepsy.

Of the 30 children under consideration, 27 developed normally prior to the onset of the disease, while three presented with developmental delays antecedent to the manifestation of symptoms. Subsequent to the onset of the conditions, eight (26.7%) children exhibited progress in both mental and motor development; nonetheless, their progress remained comparatively behind that of their unaffected peers. Video EEG showed epileptiform discharges in 25 cases, including eight cases with widespread and multifocal discharges, as well as four cases with focal discharges. Moreover, abnormal cranial MRI findings were detected in six cases.

### 3.3 Clinical treatment effectiveness

Therapeutic interventions were diversified within the 30 children. Ten were treated with a single type of anti-seizure medication (ASM), while 20 were treated with a combination of two or more ASMs. Additionally, four children adopted a ketogenic diet as part of their treatment regimen. Following the administration of ASM, 11 cases were clinically controlled with a period of at least 6 months without any epileptic attack. Furthermore, a notable reduction (≥50%) regarding to the frequency of seizures were observed in 11 cases, representing a total of 22 with effective treatment. Eight cases exhibited either ineffective treatment characterized by a decrease in the number of seizures by less than 50%, or demonstrated a progression of the disease.

The 30 children were divided into the “effective group” and the “ineffective group” according to the clinical treatment effectiveness. Comparisons revealed significant differences between the two groups in terms of pathogenic levels of ACMG, mutation type (gene) and age groups of onset (all *p* < 0.05), while there were no differences in sex, mutation source, presence or absence of status epilepticus, and efficacy of ion channels (all *p* > 0.05) (see Table 2).

**Table 2:** Single factor analysis about curative effect.

Item	Effective group	Ineffective group	*χ* ^2^	*p*-value
	*n* = 22	*n* = 8		
**Sex**			0.682	0.682
Male	12	3		
Female	10	5		
**Mutation type (gene)**			5.568	0.034
synonymous	16	2		
Non-synonymous	6	6		
Mutation source			2.7	0.155
*De novo*	14	7		
Parental generation	6	0		
**ACMG classification**			8.342	0.017
Pathogenic	9	8		
Likely pathogenic	6	0		
Uncertain significance	7	0		
**Onset age**			5.455	0.029
≤12 months	12	8		
>12 months	10	0		
**Status epilepticus**			3.519	0.101
Yes	8	6		
No	14	2		
**Ion channels**			0.085	1.000
Yes	15	5		
No	7	3		

### 3.4 Comparison of clinical features between genetically positive and negative patients

Of the 61 children who met the inclusion criteria and underwent WES, 30 (49.2%) had positive genetic findings and 31 (50.8%) had negative results. The clinical characteristics of the 31 genetically negative patients are summarized as follows: the negative group comprised 17 males (54.8%) and 14 females (45.2%), with ages ranging from 1 to 9 years. The age at seizure onset ranged from 6 months to 4 years, with 8 cases (25.8%) occurring within the first year of life. Eleven patients (35.5%) had a family history of febrile seizures, and two (6.5%) had a family history of epilepsy. The final diagnoses in the negative group included GEFS+ (10 cases, 32.2%), focal epilepsy (3 cases, 9.7%), and other types of epilepsy (18 cases, 58.1%). Developmental delay prior to seizure onset was present in two cases (6.5%), and 27 cases (87.1%) showed epileptiform discharges on EEG. Abnormal MRI findings were detected in three cases (9.7%).

A comparison of key clinical features between the genetically positive and negative groups is presented in Table 3. Patients in the positive group had a significantly earlier age at onset (≤12 months: 66.7% vs. 25.8%, *p* < 0.05), a higher frequency of status epilepticus (46.7% vs. 16.1%, *p* < 0.05), and a higher rate of developmental delay after onset (56.7% vs. 12.9%, *p* < 0.001) compared to the negative group. No significant differences were observed between the two groups regarding sex distribution, family history of febrile seizures or epilepsy, or patterns of EEG epileptiform discharges (83.3% vs. 87.1%, *p* = 0.68) or MRI abnormalities (20.0% vs. 9.7%, *p* = 0.26).

**Table 3:** Comparison of clinical features between genetically positive and negative patients.

Clinical feature	Positive group (*n* = 30)	Negative group (*n* = 31)	*χ* ^2^	*p*-value
Sex (Male/Female)	15/15	17/14	0.15	0.70
Age at onset ≤12 months	20 (66.7%)	8 (25.8%)	10.23	0.001
Family history of febrile seizures	10 (33.3%)	11 (35.5%)	0.03	0.86
Family history of epilepsy	4 (13.3%)	2 (6.5%)	0.81	0.37
Status epilepticus	14 (46.7%)	5 (16.1%)	6.58	0.01
Developmental delay after onset	17 (56.7%)	4 (12.9%)	12.84	<0.001
EEG epileptiform discharges	25 (83.3%)	27 (87.1%)	0.17	0.68
Abnormal MRI findings	6 (20.0%)	3 (9.7%)	1.27	0.26

## 4 Discussion

The rapid development of sequencing technology and its wide application in the medical field in the past decade have facilitated investigations into the association between genetic mutations and the onset of epilepsy, thereby contributing to an improved understanding of epilepsy pathogenesis (8–10). In the present study, we discovered mutations in multiple genes underlying 30 children with epilepsy related to fever sensitivity. Notably, mutations in *SCN1A*, *PCDH19*, and *ADGRV1* were identified as the principal contributors to the onset of the disease. Significantly, a nonsense mutation in the *KIAA2022* gene (c.2667G>A, p.W889X) was identified as a novel pathogenic mutation to cause febrile-sensitive epilepsy. We also revealed the variations in clinical efficacy across different pathogenicity levels as per ACMG guidelines, as well as variations across distinct age groups of onset (≤1 year group). Furthermore, we also found that children with mutations in *SCN1A* and *PCDH19* are more likely to exhibit status epilepticus compared to those with mutations in *ADGRV1*, *SCN2A*, and the calcium channel gene *PRRT2*. Therefore, genetic testing may hold promise for the early detection of factors influencing the condition in infants aged ≤1 year, thereby enhancing treatment precision and individualization.

*SCN1A* mutations are closely related to DS and GEFS+. A noteworthy subset of DS-related pathogenic genes comprises *SCN2A*, *CHD2*, *GABRA1*, *STXBP1*, and *SCN1A*, collectively responsible for 80% of the reported mutations (11), with approximately 90% are *de novo*. Among the children with DS included in this study, all 11 cases were caused by pathogenic mutations in the *SCN1A* gene. Of these, nine cases manifested *de novo* mutations, while the origin of mutation in the rest two remained unidentified, but speculated to be de novo. Parents of these two with SCN1A mutations exhibited no historical incidence of seizures, yet refused Sanger sequencing due to personal reasons. Cetica et al. (12) found that among children with *SCN1A* mutations, the risk of developing DS was 85% for those with an onset age of 0–6 months, 51% for those with an onset age of 6–12 months, and 0% for those with an onset age greater than 12 months. The application of an onset age of 6 months as a predictive indicator for DS demonstrated a sensitivity of 83.3% and specificity of 76.6%. Moreover, an analysis of the clinical characteristics of 138 children with DS by Xu et al. (13) recommended considering a DS diagnosis for children experiencing convulsive seizures induced by fever before the age of 1 year, especially for those with two or more complex febrile seizures. Therefore, in clinical practice, heightened suspicion for DS is advised for children with an onset within 6 months of age, those who experience ≥2 prolonged focal seizures or status epilepticus before the age of 1 year, or those transitioning to non-febrile seizures before 24 months of age. Consequently, comprehensive genetic screening and early clinical intervention are recommended to improve the long-term outcomes.

The *PCDH19* gene, linked to epilepsy, is located at Xq22.1 and has a unique X-linked inheritance pattern. The disease manifests in female heterozygotes or male mosaics, while male hemizygotes remain unaffected. Hundreds of *PCDH19* gene mutations have been documented, with the first exon as the primary mutation site. A minority of patients may have deletions or duplications affecting the entire gene or parts of it, predominantly arising as *de novo* mutations (14, 15). All four cases, who were all girls in our study were identified as de novo mutations, comprising one case with missense mutation, two cases with frameshift mutation, and one with deletion of the entire gene. Three of the above-mentioned mutations were not reported before. All four patients exhibited typical characteristics of cluster seizures and febrile sensitivity. In two cases, the frequency of seizures reduced significantly with age, aligning with the reported characteristics of *PCDH19*-related epilepsy (16).

Homozygous mutations in the *ADGRV1* gene can lead to severe Usher Syndrome, while heterozygous truncating mutations are closely associated with Juvenile Myoclonic Epilepsy and Febrile Seizures, typically linked to a good prognosis (17, 18). Li et al. (19) revealed that missense mutations in *ADGRV1* had a significant association with epilepsy. Most cases present in infancy and exhibit febrile sensitivity, with some patients experiencing varying degrees of developmental delay (17, 18). In the present study, all three cases with *ADGRV1* mutations were characterized by missense mutations, exhibiting similar clinical phenotypes. One of them experienced status epilepticus, a positive response to clinical treatment was observed. The origins of these mutations were not conclusively determined, yet neither parent showed clinical symptoms, which is consistent with the incomplete dominant inheritance pattern of the *ADGRV1* gene.

In this study, we identified five cases of calcium ion-related gene mutations, including mutations in *CACNA1A*, *CACNA1E*, *CACNB4*, as well as one EMAS case with a heterozygous missense mutation of the *CACNA1H* gene. *CACNA1H* encodes the T-type calcium channel, implicated in various types of epilepsy, including febrile seizures plus, childhood absence epilepsy, and myoclonic epilepsy. However, reports of such mutations in EMAS are relatively rare (20, 21). In this case, the child with *CACNA1H* mutation presented myoclonus, atonia, and absence seizures, with the disease manifesting initially as febrile seizures. This observation indicates a propensity for thermal sensitivity in calcium channels. Lamotrigine, an inhibitor of T-type calcium channels surpassing sodium valproate, was effective in this case. Additionally, epilepsy was well controlled in one case with *CACNA1E* mutation and one with *CACNA1A* mutation. To date, *CACNB4*, which encodes the β4 subunit of voltage-gated calcium channels (22) has not been associated with fever-sensitive epilepsy. In our study, we found that one case with a deletion of exon 1–14 of the *CACNB4* gene showed characteristics of fever-sensitive epilepsy after the age of 1 year, accompanied by developmental delay. Another case with a heterozygous missense in the *CACNB4* gene, displayed a predisposition to status epilepticus. These two mutations have not been reported previously, thus, significantly broaden the genetic spectrum associated with fever-sensitive epilepsy.

Epilepsy patients with *CHD2* gene mutations often present multiple types of seizures, and a few may undergo status epilepticus. Notably, mutations in this gene are common in patients with onset ages of 6 months to 4 years, with fever serving as a common trigger. In our study, the patient with *CHD2* mutation showed clinical characteristics of epilepsy related to fever sensitivity at the age of 2, with normal developmental progress. In accordance with previous reports, management with oxcarbazepine and lamotrigine yielded partial control of the condition (23–25).

The *PRRT2* gene mutations are known to be often associated with benign familial infantile epilepsy, paroxysmal exercise-induced dyskinesia, and paroxysmal choreoathetosis infantile convulsions, with only a minority experiencing simple febrile seizures (26, 27). In our study, one child carried a novel total repeat of *PRRT2*. The child developed normally and the epilepsy was well controlled with oral levetiracetam treatment.

Although early-onset epileptic encephalopathy type 32 caused by *KCNA2* gene mutation is rare (28), our study identified a case of epileptic encephalopathy attributed to a novel mutation in the *KCNA2* gene. The patient exhibited an early onset of the disease, and the epileptic seizures of various types that were significantly linked to fever. The child also showed severe encephalopathy symptoms throughout the course of the disease. While drug control improved with the patient’s age, intellectual disability persisted. Notably, the intellectual disability was significantly improved after treatment with methylprednisolone, which is the first reported case of effective hormone treatment for *KCNA2* gene-related epileptic encephalopathy. However, the correlation between the therapeutic effect and the mutation site necessitates further verification through additional samples.

*KIAA2022* plays an important role in the early development of the brain, and mutations in this gene have been reported to cause severe intellectual disability in male patients (29). De Lange et al. (30) found that 12 out of 14 female children with *KIAA2022* mutation showed refractory seizures and less severe intellectual disability than males. In our study, we identified a novel nonsense mutation in the *KIAA2022* gene, which is pathogenic according to the ACMG guideline. Intriguingly, the male patient in this case showed relatively normal intellectual development, albeit presenting initially with generalized tonic–clonic seizures during episodes of fever or low fever. Later, he had absence seizures and video EEG examinations showed multifocal and widespread epileptic discharges. Clinically, oral administration of sodium valproate and lamotrigine, complemented by diazepam during fever episodes proved effective. Thus, our findings indicate *KIAA2022* as a novel candidate gene related to febrile-sensitive epilepsy, though further confirmation through comprehensive clinical and functional studies is warranted.

A key strength of this study is the inclusion and comparative analysis of both genetically positive and negative patients from the same initial cohort of 61 children presenting with fever-sensitive seizures. Our comparison revealed that early age at onset (≤12 months), the occurrence of status epilepticus, and the development of developmental delay after seizure onset were significantly more common in children with identifiable genetic mutations. These clinical red flags may help clinicians prioritize genetic testing in resource-limited settings.

In conclusion, epilepsy related to fever sensitivity in children is primarily characterized by DS and PCDH19-related epilepsy, with prominent contributions from mutations in SCN1A, PCDH19, and ADGRV1. Furthermore, while the confirmation of our findings necessitates further extensive clinical and functional investigations involving larger sample sizes, the discovery of the novel mutation in the *KIAA2022* gene contributes to the enrichment of the gene spectrum associated with fever-sensitive epilepsy. Thus, our study provides important evidence for the early diagnosis and precise treatment of epilepsy related to fever sensitivity in children by elucidating the clinical characteristics and their correlation with gene mutations. Nevertheless, as a retrospective study with a relatively small sample size and variable timing of genetic assessment, our findings require validation through prospective studies with larger, standardized cohorts and complete follow-up to establish definitive genotype–phenotype correlations.

## Data Availability

The original contributions presented in the study are included in the article/supplementary material, further inquiries can be directed to the corresponding authors.
